# News and Events of the Week

**Published:** 1910-07-02

**Authors:** 


### News and Events of the Week.

The King has been pleased to make certain appointments on the occasion of the day set apart for the celebration of his late Majesty's birthday, from which we publish those of interest to the medical profession :—

To be a Baron of the United Kingdom : The Right Hon. Sir Walter B. Foster.—For twenty-four years Professor of Medicine at Queen's College, Birmingham.

To be a Baronet of the United Kingdom : Francis Henry Champneys, Esq., M.D.—Born in 1848; a physician to London hospitals, chairman of the Central Midwives Board, and a distinguished specialist in obstetric medicine.

To receive the honour of Knighthood : Henry S. Lunn, Esq., M.D.—Founded the Grindelwald Conference, 1892-6, and arranged the recent international municipal visits between England and Germany; Liberal candidate for Boston at the last election.

David Caldwell M'Vail, Esq., M.D.—Crown member for Scotland of the General Medical Council; physician to Glasgow Royal Infirmary.

John Lentaigne, Esq.—President of the Royal College of Surgeons, Ireland; surgeon to household of the Lord-Lieutenant.

Robert Michael Simon, Esq., M.D.—Physician to the Birmingham General Hospital; formerly Professor of Medical Jurisprudence at Mason College.

George Hastings, Esq., M.D.—Physician to Westminster General Dispensary, house physician to St. Bartholomew's Hospital, and medical officer to the Westbourne Dispensary.

Arthur Henry Downes, Esq., M.D.—Senior Medical Inspector for Poor Law Purposes to the Local Government Board; educated at Aberdeen University, formerly Medical Officer of Health in the Chelmsford and Maldon rural districts, and Acting Resident Medical Officer to University College Hospital.

To be a Knight Commander of the Most Honourable Order of the Bath : Inspector-General of Hospitals and Fleets James Porter, C.B., M.D., K.H.P.

To be Companions of the Order : Surgeon-General Arthur Thomas Sloggett, C.M.G., Principal Medical Officer, India; Surgeon-General Owen Edward Pennefather Lloyd, V.C., Principal Medical Officer, South Africa.

### Indian Honours List.

To be a Companion of the Order of the Star of India : Surgeon-General Charles Pardey Lukis, M.D., F.R.C.S., Honorary Surgeon to the Viceroy, Director-General Indian Medical Service, and an Additional Member of the Council of the Governor-General for making laws and regulations.

To be a Companion of the Order of the Indian Empire : Colonel Roderick Macrae, M.B., Indian Medical Service (retired), lately Inspector-General of Civil Hospitals, Bengal.

To receive the honour of Knighthood : Surgeon-Lieut.-Colonel Warren Roland Crooke-Lawless, C.I.E., M.D., Coldstream Guards, Surgeon to his Excellency the Viceroy.

To receive the Kaisar-I-Hind Gold Medal : Captain Robert McCarrison, M.D., Indian Medical Service, Agency Surgeon, Gilgit; Dr. T. L. Pennell, M.D., B.Sc., F.R.C.S., Medical Missionary Church Missionary Society, Bannu, North-West Frontier Province.

### Colonial Honours List.

To be Companions of the Order of St. Michael and St. George : John Gunion Rutherford, Esq., Veterinary Director-General and Live Stock Commissioner, Department of Agriculture, Canada; Aubrey Dallas Percival Hodges, Esq., M.D., Principal Medical Officer, Uganda Protectorate, in recognition of services in the suppression of sleeping sickness.

### Territorial Force.

To be Surgeon and Physician to the King : Colonel Andrew Clark, F.R.C.S., Administrative Medical Officer 2nd London Territorial Division, to be Honorary Surgeon to the King; Colonel Joseph W. Blandford, Administrative Medical Officer Northumbrian Territorial Division, to be Honorary Physician to the King.

---

His Majesty the King, her Majesty the Queen, her Majesty Queen Alexandra, and his Royal Highness the Prince of Wales have graciously accepted copies of the new Prayer-book from Messrs. Eyre and Spottiswoode.

A party of about twenty American surgeons having arrived in England from the principal hospitals of the United States, organised by the American Society of Clinical Surgery, is to inspect surgical operations at the London hospitals and elsewhere.

The Special Board for Medicine of the University of Cambridge has recommended that the degree of M.C. be conferred on Mr. Herbert J. Paterson, M.A., M.B., B.C., F.R.C.S. Eng., of Trinity College, without examination, in consideration of his contributions to the science and art of surgery.

At an examination in sanitary science as applied to buildings and public works, held in Leeds on June 10 and 11, 1910, by the Royal Sanitary Institute, four candidates presented themselves, of whom one was successful. At the examination in hygiene in its bearing on school life, ten candidates presented themselves for the certificate, and three candidates were granted certificates, while three other candidates were successful in Part I. only. At the examination for women health visitors and school nurses, five candidates were successful.

The Society of Apothecaries of London has addressed a memorial to the Lord President of the Council upon the subject of the Midwives Bill, 1910. It is there urged that before the Privy Council abolishes the power of appointment of a representative on the Midwives Board by any body or person, the body or person in question should have the right of being heard before the Council. It is also suggested that payment of the travelling expenses of the members of the Board should be made obligatory.

The King held a Court on Wednesday, June 22, at St. James's Palace, when the following deputations from privileged bodies were separately introduced into the Presence by the Lord Chamberlain: From the Royal College of Physicians of London, introduced by Sir Thomas Barlow, Bart., President, who presented Sir William Allchin and Dr. Kingston Fowler. From the Royal College of Physicians of Edinburgh, introduced by Dr. W. Allan Jamieson, the President, who presented Dr. John Playfair and Dr. Norman Walker. From the Royal College of Surgeons of Edinburgh, introduced by Mr. Joseph Montagu Cotterill, the President, who presented Mr. Charles McGillivray and Mr. Robert Johnston.

Recently, before Mr. Justice Darling and a special jury, Dr. Hooper, a medical man who for many years had been in practice at Sutton, sued the proprietors of the *Sutton Herald* for libel. The plaintiff was chairman of the Sutton Urban District Council, and was on its Sanitary Committee. In connection with the council was an isolation hospital at Cuddington. A controversy arose as to the sending out from the isolation hospital of a little girl who had scarlet fever. Mr. Justice Darling suggested that the parties should confer with him with a view to a settlement, and in the result the defendants most emphatically recognised that there was no justification for any imputation. They were unable to explain how the name of the plaintiff came to be inserted in the paper by someone in their office, but they felt that they were to blame for the unfortunate mistake, and were therefore willing to pay the plaintiff's costs.

The New Palace Steamers announce that, in reply to numerous inquiries they have received, on Friday, July 1, and on every following Friday during the season, their P.S. "Royal Sovereign" will leave Old Swan Pier at 9 a.m. for Southend, Margate, and Ramsgate, thus making a daily service to those ports.

Messrs. Blacks' colour books leave few countries of the world undepicted; but Russia has not yet found a place in the series. This omission is to be made good by the publication, in the first place, of a volume on "St. Petersburg," painted by F. de Haenen, and described by G. Dobson. Other Russian centres will follow later.

The Birmingham Education Committee has sent to the Prime Minister a memorandum stating that mentally defective children who have attended special schools cannot be withdrawn from public control at the age of sixteen years without grave danger and injury to the national welfare. The committee hopes that the Government will at the earliest possible time introduce legislation according with the recommendations of the Royal Commission on the Care and Control of the Feeble-minded.

Mr. W. H. Lever, chairman of the Liverpool School of Tropical Medicine, gave a complimentary banquet last Saturday at Liverpool to some of the recipients of the Mary Kingsley Medal of the Society of Tropical Medicine and Hygiene. Mr. Lever said that at 11 o'clock that night the third expedition of the school left for Malta under the charge of Mr. Newstead. They had two other expeditions already at work—one under Sir Rubert Boyce in West Africa, and another under Dr. Thomas in Brazil. Surgeon-General Sir Alfred Keogh, C.B., said that his special mission had been to make the War Office realise the fact that medicine was an applied science, and that it was not concerned merely with the treatment of disease, but with its prevention. They could make such countries as West and Central Africa as salubrious as Liverpool if they would only take the trouble to think about it and would organise for the purpose. Thanks to applied medicine, there had been added to the Indian Army for defensive purposes last year the equivalent of two battalions. After Sir David Bruce, Professor Ross, C.B., and Dr. Sandwith also had spoken, the company drank to the memory of the late Sir Alfred Jones, founder of the school.

Lord Sanderson presided at a meeting of the C.O.S. at which Dr. Grey, Chairman of the Medical Advisory Committee, delivered an address on the reports of district committees on crèches. The reports, he said, showed that little precaution was taken as a rule to prevent the admission of unhealthy children or the spread of infection. Medical supervision, as a rule, meant nothing more than that there was a doctor who could be sent for if necessary. Crèches were not subject to inspection by any authority, and were governed only by the general provisions of the Public Health Act. Resolutions were passed expressing the opinion that all crèches should be registered and subject to inspection.

The provincial sessional meeting of the Royal Sanitary Institute will be held on Saturday, July 16, in the New Medical Schools, Downing Street, Cambridge, at 11 a.m., when a discussion will take place on "The Sterilisation of Water by Chlorine and Ozone," to be opened by Professor G. Sims Woodhead. Tickets for admission of visitors may be had on application to Dr. Bushell Anningson, Walt Ham Sal, Barton Road, Cambridge, who is kindly acting as the hon. local secretary to the meeting. After the meeting there will be a luncheon in the Hall of Gonville and Caius College (by kind permission of the Master and Fellows), and at 2.15 p.m. brakes will be in readiness at Senate House Hill to convey members to the waterworks at Fulbourn, where the chlorine system is at work.

